# Antioxidant and antimicrobial activities of ethyl acetate extract, fractions and compounds from stem bark of *Albizia adianthifolia* (Mimosoideae)

**DOI:** 10.1186/1472-6882-12-99

**Published:** 2012-07-18

**Authors:** Jean de Dieu Tamokou, Deke James Simo Mpetga, Paul Keilah Lunga, Mathieu Tene, Pierre Tane, Jules Roger Kuiate

**Affiliations:** 1 Department of Biochemistry, Laboratory of Microbiology and Antimicrobial Substances, Faculty of Science, University of Dschang, PO Box 67, Dschang, Cameroon; 2 Department of Chemistry, Laboratory of Natural Product Chemistry, Faculty of Science, University of Dschang, PO Box 67, Dschang, Cameroon; 3 Department of Biochemistry, Laboratory of Phytobiochemistry and Medicinal Plant Study, Faculty of Science, University of Yaounde I, PO Box 812, Yaounde, Cameroon

**Keywords:** *Albizia adianthifolia*, Antioxidant, Antimicrobial, Phenols, Fractionation, Aurantiamide acetate

## Abstract

**Background:**

*Albizia adianthifolia* is used traditionally in Cameroon to treat several ailments, including infectious and associated diseases. This work was therefore designed to investigate the antioxidant and antimicrobial activities of ethyl acetate extract, fractions and compounds isolated from the stem bark of this plant.

**Methods:**

The plant extract was prepared by maceration in ethyl acetate. Its fractionation was done by column chromatography and the structures of isolated compounds were elucidated using spectroscopic data in conjunction with literature data. The 1,1-diphenyl-2-picrylhydrazyl (DPPH) and trolox equivalent antioxidant capacity (TEAC) assays were used to detect the antioxidant activity. Broth micro-dilution method was used for antimicrobial test. Total phenol content was determined spectrophotometrically in the extracts by using Folin–Ciocalteu method.

**Results:**

The fractionation of the extract afforded two known compounds: lupeol (**1**) and aurantiamide acetate (**2**) together with two mixtures of fatty acids: oleic acid and *n*-hexadecanoic acid (B_1_); *n*-hexadecanoic acid, octadecanoic acid and docosanoic acid (B_2_). Aurantiamide acetate was the most active compound. The total phenol concentration expressed as gallic acid equivalents (GAE) was found to vary from 1.50 to 13.49 μg/ml in the extracts. The antioxidant activities were well correlated with the total phenol content (*R*^*2*^ = 0.946 for the TEAC method and *R*^*2*^ = 0.980 for the DPPH free-radical scavenging assay).

**Conclusions:**

Our results clearly reveal that the ethyl acetate extract from the stem bark of *A. adianthifolia* possesses antioxidant and antimicrobial principles. The antioxidant activity of this extract as well as that of compound **2** are being reported herein for the first time. These results provide promising baseline information for the potential use of this plant as well as compound **2** in the treatment of oxidative damage and infections associated with the studied microorganisms.

## Background

*Albizia adianthifolia* (Schumach) W. F. Wight (Mimosoideae), also known as *A*. *chirindensisA*. *fastigiata*, is a big tree found in moist and tropical forest zones as well as areas that are transitional to woodland [1]. This plant is used in Central and West Africa for the treatment of skin diseases, bronchitis, inflamed eyes, tapeworm, headaches and sinusitis [2,3]. Various parts of *A. adianthifolia* are traditionally used to treat different diseases. The maceration of stem bark and root is used as an antidote against poison or applied in pomade on inflamed eye; the decoction of stem bark is drunk in the treatment of abdominal pains, typhoid fever and infections of urinary and respiratory tracts. Some biological activities exhibited by *A. adianthifolia* have been documented. Ethanolic extract of the root of this plant showed *in vitro* immunomodulatory activity on the Jurkart T cell line and haemolytic property against sheep erythrocytes [4]. These activities were attributed to the triterpenoidal saponins contained in this extract [4]. *A. adianthifolia* has also been reported to contain several flavonoids [5]. The root extracts of *A. adianthifolia* showed antibacterial, anti-inflammatory and anti-cholinesterase effects [6]. In many developing countries, the most infectious diseases are of microbial origin. With the advent of ever-increasing resistant bacterial and yeast strains, there has been a corresponding rise in the universal demand for natural antimicrobial therapeutics [7,8]. Microbial infections, especially due to *StaphylococcusStreptococcus* and *Pseudomonas* species, and the presence of oxygen free radicals, are known impediments to wound healing [9]. Any agent capable of eliminating or reducing the number of microorganisms present in a wound, as well as reducing the level of reactive oxygen species (ROS), may facilitate the wound healing process. It then becomes necessary to search for new antimicrobial and antioxidant drugs, especially those that would be cheap and thus easily affordable by poor population. The present work was therefore designed to investigate the antioxidant and antimicrobial activities of ethyl acetate extract, fractions and compounds isolated from the stem bark of *A. adianthifolia.*

## Materials and methods

### Plant material

The stem bark of *A. adianthifolia* was collected from Mbouda (West Region of Cameroon) in January 2010. The plant material was identified at the Cameroon National Herbarium in Yaoundé where a voucher specimen (N° 19778/SRFCam) was deposited.

### Extraction, fractionation and isolation

The stem bark of *A. adianthifolia* was dried at room temperature (25 ± 2°C) for three weeks and crushed. Four kilograms of obtained powder was macerated into 15 l ethyl acetate (Merck) for two days and this process was repeated twice. After filtration, the filtrate was evaporated to dryness at 50°C under reduced pressure using a rotary evaporator. The dried crude extract (1.75% w/w) was stored at +4°C. A portion of 60 g of crude extract was then subjected to column chromatography (22 cm x 8 cm column) using 300 g of silica gel 40 (particle size 0.2-0.5 mm). The column was successively eluted with hexane (4200 ml), Hexane – EtOAc [19 : 1 (3900 ml), 4 : 1 (4200 ml), 7 : 3 (4800 ml), 3 : 2 (3300 ml), 1 : 1 (3300 ml) and 3 : 7 (6600 ml)] mixtures, ethyl acetate (4500 ml), ethyl acetate-methanol [19 : 1 (3000 ml), 17 : 3 (3000 ml) and 7 : 3 (1500 ml)] mixtures and methanol (3900 ml). One hundred and fifty four fractions of 300 ml each were collected and combined on the basis of their thin layer chromatography (TLC) profiles to afford nine main fractions. Fractions 1–17, 18–25, 26–40, 41–54, 55–78, 79–92, 93–124, 125–148 and 149–160 were referred to as F_1_, F_2_, F_3_, F_4_, F_5_, F_6_, F_7_, F_8_ and F_9_ respectively. These fractions were tested for their antimicrobial/antioxidant activities and the most active fractions were further subjected to purification in order to isolate the active principles. Fraction F_2_ (2.80 g) was loaded on a silica gel column (0.063-0.20 mm, 120 g) eluted with hexane-EtOAc gradients and 37 subfractions of 100 ml each were collected. Subfractions 1–7 obtained with hexane were purified on a sephadex LH-20 column eluted with CH_2_Cl_2_-MeOH (9:1) to afford lupeol (45 mg) as yellow crystal. Subfractions 8–20 obtained with hexane-EtOAc (9:1) were purified by CC on sephadex LH-20 gel eluted with hexane-EtOAc (8:2) to give the mixture of fatty acids B_1_ (33 mg): oleic acid and *n*-hexadecanoic acid as yellowish crystal. Subfractions 21–32 obtained with hexane-EtOAc (1:1) were purified on a sephadex LH-20 column eluted with hexane-EtOAc (7:3) to yield the mixture of fatty acids B_2_ (46 mg): *n*-hexadecanoic acid, octadecanoic acid and docosanoic acid as yellowish crystal. Aurantiamide acetate (30 mg) was obtained from fraction F_4_ (17.20 g, eluted with CH_2_Cl_2_-EtoAc 19:1) after purification by preparative TLC. Fraction F_5_ (10.30 g) yielded two individual minor compounds (detected only on TLC) and a complex mixture.

### Identification of the isolated compounds

The structures of the isolated compounds were established using spectroscopic analysis, especially, NMR spectra in conjunction with 2D experiments and by direct comparison with published information [10,11] and authentic specimens obtained in our laboratory for some cases. Melting points (uncorr.) were determined on a Kofler apparatus. ^1^ H, 2D ^1^ H-^1^ H COSY, ^13^ C, 2D HMQC and HMBC spectra were recorded with a Bruker Avance 500 MHz spectrometer. Optical spectra were recorded with a NICOLET 510 P FT-IR spectrometer, a UV-2101 PC spectrometer, and a Perkin- Elmer 241 polarimeter. Column chromatography was run on Merck silica gel 60 and gel permeation on sephadex LH-20, while TLC were carried out either on silica gel GF_254_ pre-coated plates (analytical TLC) or on silica gel 60 PF_254_ containing gypsum (preparative TLC), with detection accomplished by spraying with 50% H_2_SO_4_ followed by heating at 100°C, or by visualizing with an UV lamp at 254 and 366 nm.

The mixtures of fatty acids were identified by comparison of their mass spectra with those available from the equipment database (Wiley 7 Nist 05.L) and from the literature. Gas chromatography–mass spectrometry (GC-MS) data were obtained with an Argilent 6890 N Network GC system/5975 Inert x L Mass selective Detector at 70 eV and 20°C. The GC column was a CP- Sil 8 CB LB, fused silica capillary column (0.25 mm x 30 m, film thickness 0.25 μm). The initial temperature was 50°C for 1 min, and then heated at 10°C/min to 300°C. The carrier gas was helium at a flow rate of 1.20 ml/min. Mass spectral data were used to identify fatty acid fractions.

### Determination of total phenol content

Total phenol content was determined spectrophotometrically in the extracts by using Folin–Ciocalteu method as previously described [12]. The Folin–Ciocalteu reagent was prepared by mixing 5 g sodium tungstate, 1.25 g sodium molybdate, 2.50 ml of 85% phosphoric acid, 10 ml 20% hydrochloric acid, 7.50 g lithium sulfate, two drops of bromine and deionized water to a final volume of 50 ml. Further, stock solutions of 20% sodium carbonate and 400 mg/l gallic acid were added. For each sample, 20, 10 and 1 μl of 10 mg/ml ethanolic extract or 20 μl of 1 mg/ml ethanolic isolated compounds were added to 640 μl distilled water and 200 μl freshly prepared Folin–Ciocalteu reagent, followed by incubation in the dark for 5 min.

Then, 150 μl of 20% sodium carbonate solution were added and samples were incubated in the dark for 30 min. The solution turned deep blue. The final concentration of the tested samples in the assayed solution was 100 μg/ml and 10 μg/ml for the extracts and isolated compounds respectively. At the same time, gallic acid standards of 6.25, 12.50, 25, 50 and 75 μg/ml final concentration solutions were reacted with the Folin–Ciocalteu reagent in the same way as the samples. The UV–vis spectra of all the samples were recorded against the reference solution (zero gallic acid) and the absorbance was monitored at 725 nm. The measurements were done in triplicate. For the gallic acid standards, a calibration curve (Pearson’s correlation coefficient: *R*^2^ = 0.992) was constructed and the total level of phenolics for each sample was determined in terms of gallic acid equivalents.

### Antimicrobial assay

#### Micro-organisms

The microorganisms used in this study consisted of six bacteria (*Enterococcus faecalis* ATCC10541, *Staphylococcus aureus* ATCC25923, *Pseudomonas aeruginosa* ATCC27853, *Escherichia coli* ATCC11775, *Klebsiella pneumoniae* ATCC13883, *Salmonella typhi* ATCC6539) and seven fungi (*Candida albicans* ATCC9002, ATCC2091 and 24433, *Candida parapsilosis* ATCC22019, *C. lusitaniae* ATCC200950, *C*. *tropicalis* ATCC750, *C*. *krusei* ATCC6258); all of which are reference strains obtained from American Type Culture Collection. Also, included were two clinical isolates of bacteria (*Proteus mirabilis*, *Shigella flexneri*) collected from Pasteur Centre (Yaoundé-Cameroon) and two fungal strains (C. *glabbrata* IP35, *Cryptococcus neoformans* IP95026) obtained from Pasteur Institute (IP, Paris-France). The bacterial and yeast strains were grown at 35°C and maintained on nutrient agar (NA, Conda, Madrid, Spain) and Sabouraud Dextrose Agar (SDA, Conda) slants respectively.

#### Determination of the minimum inhibitory concentration (MIC) and minimum microbicidal concentration (MMC)

MIC was determined by broth micro dilution method as previously reported [13]. The inocula of micro-organisms were prepared from 24 h old broth cultures. The absorbance was read at 600 nm and adjusted with sterile physiological solution to match that of a 0.5 McFarland standard solution. From the prepared microbial solutions, other dilutions with sterile physiological solution were prepared to give a final concentration of 10^6^ colony-forming units (CFU) per millilitre for bacteria and 2x10^5^ spores per millilitre for yeasts. Stock solutions of the extracts (crude extract and fractions) were prepared in 5% aqueous tween 20 (Fisher chemicals) at concentrations of 50 mg/ml (for crude extract and fractions) and 1.60 mg/ml (for pure compounds). The two-fold serial dilutions in concentrations of the extracts (25–0.048 mg/ml) and pure compounds (800–0.39 μg/ml) were prepared in Mueller Hinton Broth (MHB) (Conda, Madrid, Spain) for bacteria and Sabouraud Dextrose Broth (SDB) (Conda, Madrid, Spain) for yeasts. For every experiment, a sterility check (5% aqueous tween 20 and medium), negative control (5% aqueous tween 20, medium and inoculum) and positive control (5% aqueous tween 20, medium, inoculum and water-soluble antibiotics) were included. In general, the 24-macro well plates (Nunclon, Roskilde, Danmark) were prepared by dispensing into each well 880 μl of an appropriate medium, 100 μl of test substances and 20 μl of the inoculum (10^6^ CFU/ml for bacteria and 5x10^5^ spores/ml for yeasts). The content of each well was mixed thoroughly with a multi-channel pipette and the macro-well plates were covered with the sterile sealer and incubated at 35 °C for 24 h (for bacteria) and 48 h (for yeasts) under shaking by using a plate shaker (Flow Laboratory, Germany) at 300 rpm. Microbial growth in each well was determined by observing and comparing the test wells with the positive and negative controls. The absence of microbial growth was interpreted as the antibacterial or antifungal activities. The MIC was the lowest concentration of the test substances that prevented visible growth of micro-organisms. Minimum Bactericidal Concentrations (MBCs) or Minimum Fungicidal Concentrations (MFCs) were determined by plating 10 μl from each negative well and from the positive growth control on Mueller Hinton Agar (for bacteria) and Sabouraud Dextrose Agar (for yeasts). MBCs or MFCs were defined as the lowest concentration yielding negative subcultures or only one colony. All the experiments were performed in triplicate. Gentamicin and nystatin at the concentration ranging between 400 and 0.78 μg/ml served as positive controls for antibacterial and antifungal activities respectively.

### Antioxidant assay

#### DPPH free radical scavenging assay

The free radical scavenging activity of the extracts as well as their isolated compounds was evaluated according to described methods [14]. Briefly, the test samples, prior dissolved in DMSO (SIGMA) beforehand, were mixed with a 20 mg/l 2,2-diphenyl-1-picryl-hydrazyl (DPPH) methanol solution, to give final concentrations of 10, 50, 100, 500 and 1000 μg/ml. After 30 min at room temperature, the absorbance values were measured at 517 nm and converted into percentage of antioxidant activity. L-ascorbic acid was used as a standard control. The percentage of decolouration of DPPH (%) was calculated as follows:

(1) $$\%\text{ decolouration of DPPH}=\frac{\left(\text{Absorbance of control }-\text{ Absorbance of test sample}\right)\;\text{X }100}{\text{Absorbance of control}}$$

The percentage of decolouration of DPPH (%) was plotted against the test sample. Also, the percentage of decolouration of DPPH was converted in probits. The probit values were plotted against the logarithmic values of concentrations of the test samples and a linear regression curve was established in order to calculate the EC_50_ (μg/ml), which is the amount of sample necessary to decrease by 50% the absorbance of DPPH. All the analysis were carried out in triplicate.

#### Trolox equivalent antioxidant capacity (TEAC) assay

The TEAC test was done as previously described [15] with slight modifications. In a quartz cuvette, to 950 μl acetate buffer (pH =5.0, 100 mM), the following were added: 20 μl laccase (1 mM stock solution), 20 μl test sample, 10 μl ABTS (2,2'-azinobis(3-ethylbenzothiazoline-6-sulfonic acid)) (74 mM stock solution). The sample concentrations in the assay mixture were 200, 100, 10 μg/ml for the extracts and 20 μg/ml for the isolated compounds. The content of the generated ABTS^**●+**^ radical was measured at 420 nm after 230 s reaction time and was converted to gallic acid equivalents by the use of a calibration curve (Pearson’s correlation coefficient: *R*^2^ = 0.997) constructed with 0, 4, 10, 14, 28, 56, 70 μM gallic acid standards rather than Trolox. Experiments were done in triplicate.

### Statistical analysis

Statistical analysis was carried out using Statistical Package for Social Science (SPSS, version 12.0). The experimental results were expressed as the mean ± Standard Deviation (SD). Group comparisons were performed using One Way ANOVA followed by Waller-Duncan Post Hoc test. A p value of 0.05 was considered statistically significant.

## Results

### Phytochemical analysis

**Figure 1:**
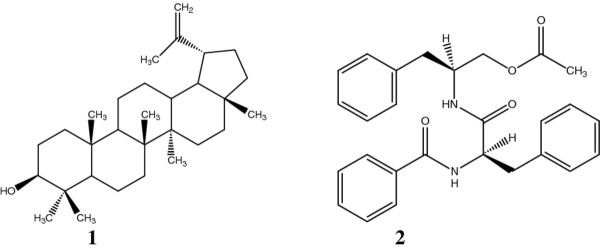
Chemical structures of lupeol (1) and aurantiamide acetate (2).

Two known compounds: lupeol (**1**) and aurantiamide acetate (**2**) and two main mixtures of fatty acids (B_1_: oleic acid and *n*- hexadecanoic acid; B_2_: *n*-hexadecanoic acid, octadecanoic acid and docosanoic acid were isolated from ethyl acetate extract of *A. adianthifolia* stem bark. The structures of compounds **1** and **2** are presented on Figure 1.

### Antimicrobial activity

In this study, the antibacterial and antifungal activities of the crude extract, fractions and the isolated compounds were evaluated. The broth micro dilution method was applied to 17 microorganisms including fungi from *Candida* and *Cryptococcus* genus, gram-positive and gram negative bacteria. The results summarized in Tables 1 and 2 showed that the crude extract, fractions F_2_-F_5_, mixtures of fatty acids B_1_ and B_2_, compounds **1** and **2** prevented the growth of most of the tested microorganisms. The MIC values obtained varied from 0.006 to 6.25 mg/ml and 0.04 to 6.25 mg/ml for yeasts and bacteria respectively (Table 1). The lowest MIC value (0.006 mg/ml) was recorded with compound **2** on *Candida parapsilosis*, *Candida tropicalis* and *Cryptococcus neoformans*. The inhibition effects of the crude extract, fraction F_4_ and compound **2** were observed on all the tested microorganisms (100%). Those of fractions F_2_, F_3_, F_5_, B_1_, B_2_ and compound **1** were respectively noted on 8 (47.5%), 9 (52.94%), 14 (82.35%), 9 (52.94%), 9 (52.94%) and 9 (52.94%) of the 17 tested microbial species. The results of the MMC determination (Table 2) also indicated a detectable MMC value within the tested interval for crude extract, fractions and compounds on several tested microorganisms. Compound **2** was the most active sample. Moreover, its MIC values were often equal to or lower than those of reference drugs gentamicin and nystatin. *Proteus mirabilis*, *Shigella flexneri*, *Staphylococcus aureus* and *Enterococcus faecalis* were in general found to be the most sensitive bacteria species while *Candida lusitaniae* and *Cryptococcus neoformans* showed the best susceptibility amongst the yeasts tested (Table 1). The MICs were generally four-fold less than the corresponding MMCs (Tables 1 and 2). The crude extract was more effective on bacteria as compare to fungi. Fractionation enhanced the antimicrobial activity of the crude extract in fraction F_4_. However, these activities decreased in fractions F_2_, F_3_ and F_5_. No activity (MIC **>**12.50 mg/ml) was noticed in fractions F_1_, F_6_, F_7_, F_8_ and F_9_ for all the microorganisms tested (not shown).

**Table 1:** **Minimum inhibitory concentrations of the crude extract, fractions and compounds isolated from** ***A. adianthifolia***

**Name of microorganism Bacteria**	**Minimum inhibitory concentrations (mg/ml)**									
	**Crude extract**	**F**_**2**_	**F**_**3**_	**F**_**4**_	**F**_**5**_	**B**_**1**_	**B**_**2**_	**1**	**2**	**References**^a^
*Pseudomonas aeruginosa*	0.19	na	/	0.19	1.56	na	na	na	0.10	0.025
*Proteus mirabilis*	0.39	3.12	1.56	0.39	0.19	0.10	0.40	0.20	0.05	0.10
*Klebsiella pneumoniae*	0.78	na	/	0.39	0.78	na	na	na	0.05	0.025
*Shigella flexneri*	0.19	6.25	6.25	0.19	0.39	0.05	0.10	0.10	0.05	0.05
*Salmonella typhi*	0.09	3.12	3.12	0.04	0.39	0.20	0.40	na	0.10	0.05
*Escherichia coli*	0.78	na	/	0.78	1.56	na	na	na	0.05	0.025
*Staphylococcus aureus*	0.09	3.12	3.12	0.09	0.78	0.20	0.80	0.20	0.05	0.05
*Enterococcus faecalis*	0.78	3.12	3.12	0.04	0.39	0.40	0.20	0.40	0.10	0.012
**Yeasts**										
*Candida albicans* ATCC 9002	1.56	na	/	1.56	3.12	0.20	0.40	0.40	0.025	0.001
*Candida albicans* ATCC 2091	3.12	na	/	0.39	0.78	/	/	/	0.025	0.0015
*Candida albicans* ATCC 24433	0.78	3.12	3.12	0.78	1.56	/	/	/	0.05	0.001
*Candida parapsilosis*	3.12	na	1.56	3.12	6.25	/	/	0.20	0.006	0.01
*Candida tropicalis*	6.25	na	na	6.25	na	0.40	0.10	/	0.006	0.006
*Candida krusei*	1.56	na	na	0.78	na	/	/	0.40	0.012	0.003
*Candida glabbrata*	1.56	na	na	1.56	na	/	/	/	0.025	0.012
*Candida lusitaniae*	0.39	1.56	1.56	0.09	0.78	0.10	0.20	0.10	0.05	0.001
*Cryptococcus neoformans*	0.78	6.25	3.12	0.39	1.56	0.20	0.40	0.20	0.006	0.001

The results are the mean values of triplicate tests measured after 24–48 h incubation at 35 ° C; na: not active; /: not tested; ^a^gentamicin and nystatin were used as reference antibiotics for bacteria and yeasts respectively; B_1:_ Oleic acid and *n*-hexadecanoid acid; B_2:_*n*-hexadecanoic acid, octadecanoic acid and docosanoic acid; **1:** lupeol; **2:** aurantiamide acetate.

**Table 2:** **Minimum microbicidal concentrations of the crude extract, fractions and compounds isolated from** ***A. adianthifolia***

**Name of microorganism Bacteria**	**Minimum microbicidal concentrations (mg/ml)**									
	**Crude extract**	**F**_**2**_	**F**_**3**_	**F**_**4**_	**F**_**5**_	**B**_**1**_	**B**_**2**_	**1**	**2**	**References**^**a**^
*Pseudomonas aeruginosa*	0.39	na	/	0.39	3.12	na	na	na	0.10	0.025
*Proteus mirabilis*	0.78	3.12	3.12	0.39	0.39	0.20	0.80	0.20	0.05	0.10
*Klebsiella pneumoniae*	1.56	na	/	0.39	1.56	na	na	na	0.05	0.025
*Shigella flexneri*	0.39	6.25	6.25	0.39	0.39	0.10	0.20	0.20	0.05	0.05
*Salmonella typhi*	0.19	3.12	3.12	0.09	0.39	0.40	0.80	na	0.10	0.05
*Escherichia coli*	1.56	na	/	1.56	1.56	na	na	na	0.05	0.025
*Staphylococcus aureus*	0.39	3.12	3.12	0.09	1.56	0.20	0.80	0.20	0.05	0.05
*Enterococcus faecalis*	0.78	3.12	3.12	0.09	0.39	0.40	0.20	0.40	0.10	0.012
**Yeasts**										
*Candida albicans* ATCC 9002	3.12	na	/	3.12	6.25	>0.80	0.80	0.40	0.025	0.001
*Candida albicans* ATCC 2091	6.25	na	/	1.56	1.56	/	/	/	0.025	0.0015
*Candida albicans* ATCC 24433	1.56	3.12	3.12	1.56	3.12	/	/	/	0.05	0.001
*Candida parapsilosis*	6.25	na	1.56	3.12	6.25	/	/	0.20	0.012	0.01
*Candida tropicalis*	6.25	na	na	6.25	na	>0.80	0.20	/	0.025	0.006
*Candida krusei*	3.12	na	na	1.56	na	/	/	0.40	0.012	0.03
*Candida glabbrata*	3.12	na	na	1.56	na	/	/	/	0.05	0.012
*Candida lusitaniae*	0.78	3.12	3.12	0.19	0.78	>0.80	0.40	0.20	0.05	0.001
*Cryptococcus neoformans*	0.78	6.25	6.25	0.78	3.25	>0.80	0.80	0.20	0.006	0.001

The results are the mean values of triplicate tests measured after 24–48 h incubation at 35 ° C; na: not active; /: not tested; ^a^gentamicin and nystatin were used as reference antibiotics for bacteria and yeasts respectively; B_1:_ Oleic acid and *n*-hexadecanoid acid; B_2:_*n*-hexadecanoic acid, octadecanoic acid and docosanoic acid; **1:** lupeol; **2:** aurantiamide acetate.

### Total phenol content

**Figure 2:**
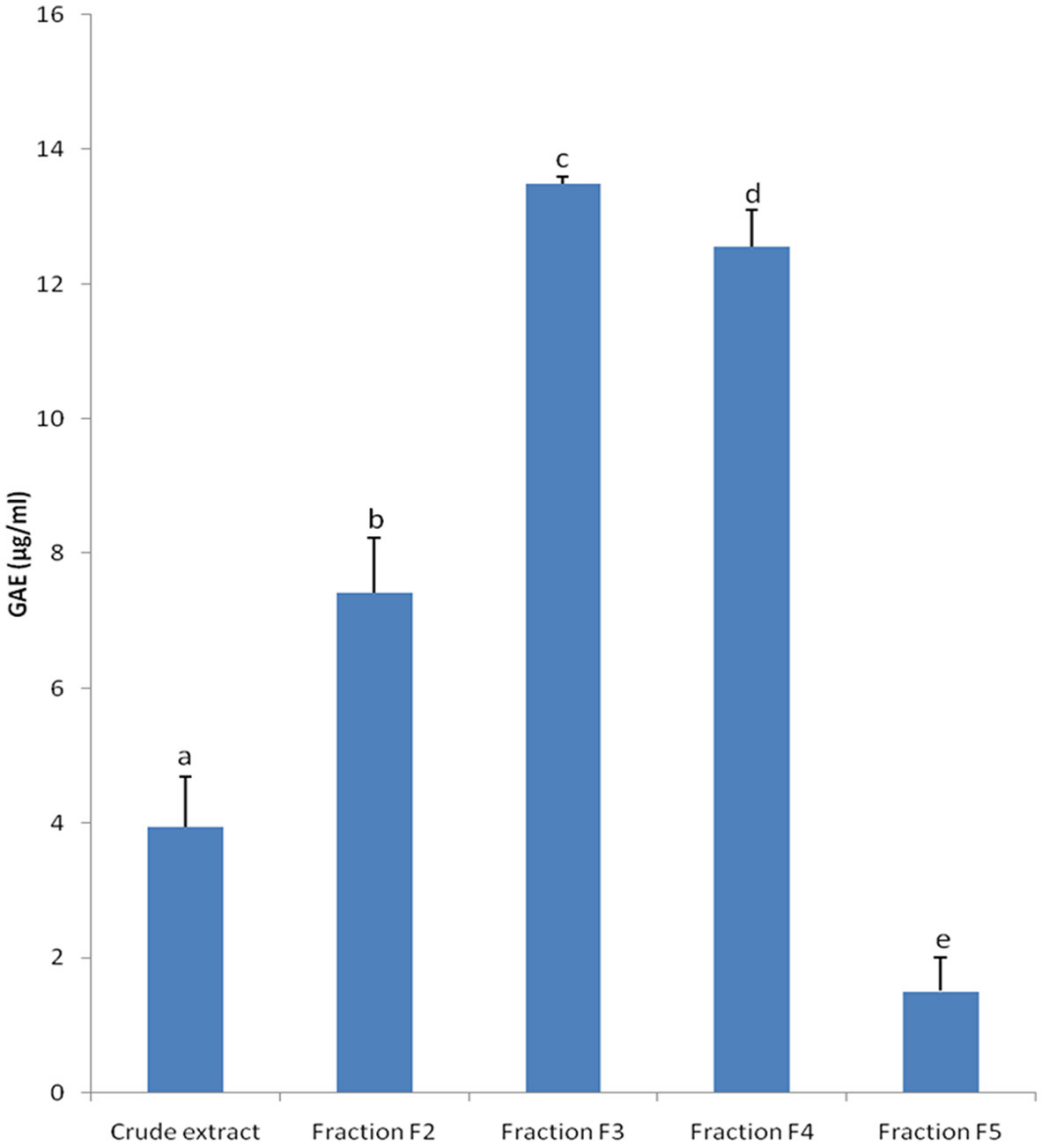
**Total phenol content expressed as gallic acid equivalents (GAE; μg/ml) in crude extract and fractions.** Bars represent the mean ± SD of three independent experiments carried out in triplicate. Letters a - e indicate significant differences between samples according to one way ANOVA and Waller Duncan test; *p* < 0.05.

The Folin-Ciocalteu assay is one of the oldest methods developed to determine the content of total phenols [16]. In this work, the total phenol content of crude extract and fractions from *A. adianthifolia* stem bark was analyzed. As shown in Figure 2, the total phenol content expressed as gallic acid equivalents (GAE) was found to vary from 1.50 to 13.49 μg/ml in the extracts. Also, the fractionation increased the total phenol content of the crude extract (GAE: 3.95 μg/ml) in fractions F_2_, F_3_ and F_4_ (GAE: 7.42, 13.49 and 12.56 μg/ml respectively); but the amount was low in fraction F_5_ (GAE: 1.50 μg/ml).

### Antioxidant activity

**Figure 3:**
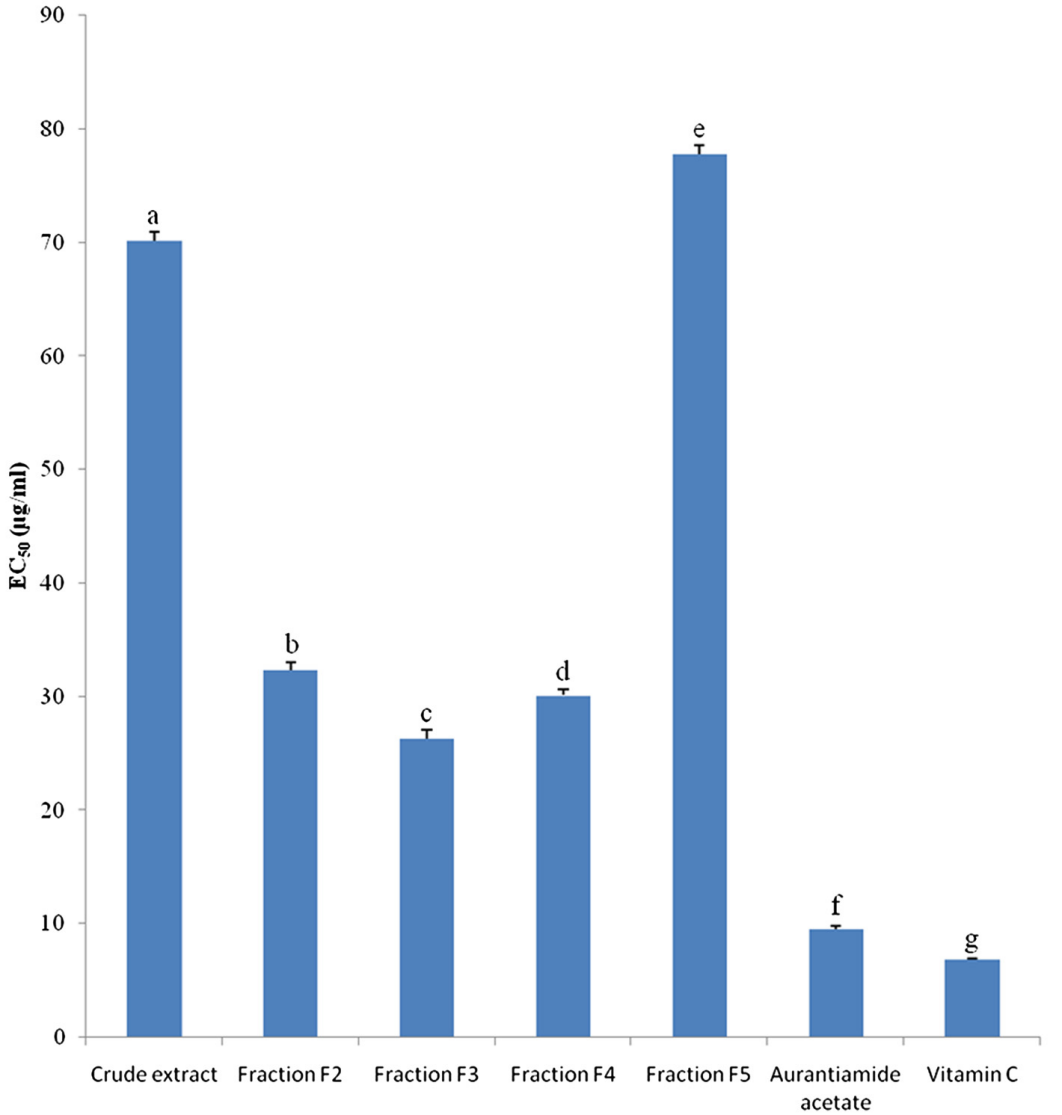
**Equivalent concentrations of test samples scavenging 50% of DPPH radical (EC**_**50**_**).** Bars represent the mean ± SD of three independent experiments carried out in triplicate. Letters a - g indicate significant differences between samples according to one way ANOVA and Waller Duncan test; *p* < 0.05.

**Figure 4:**
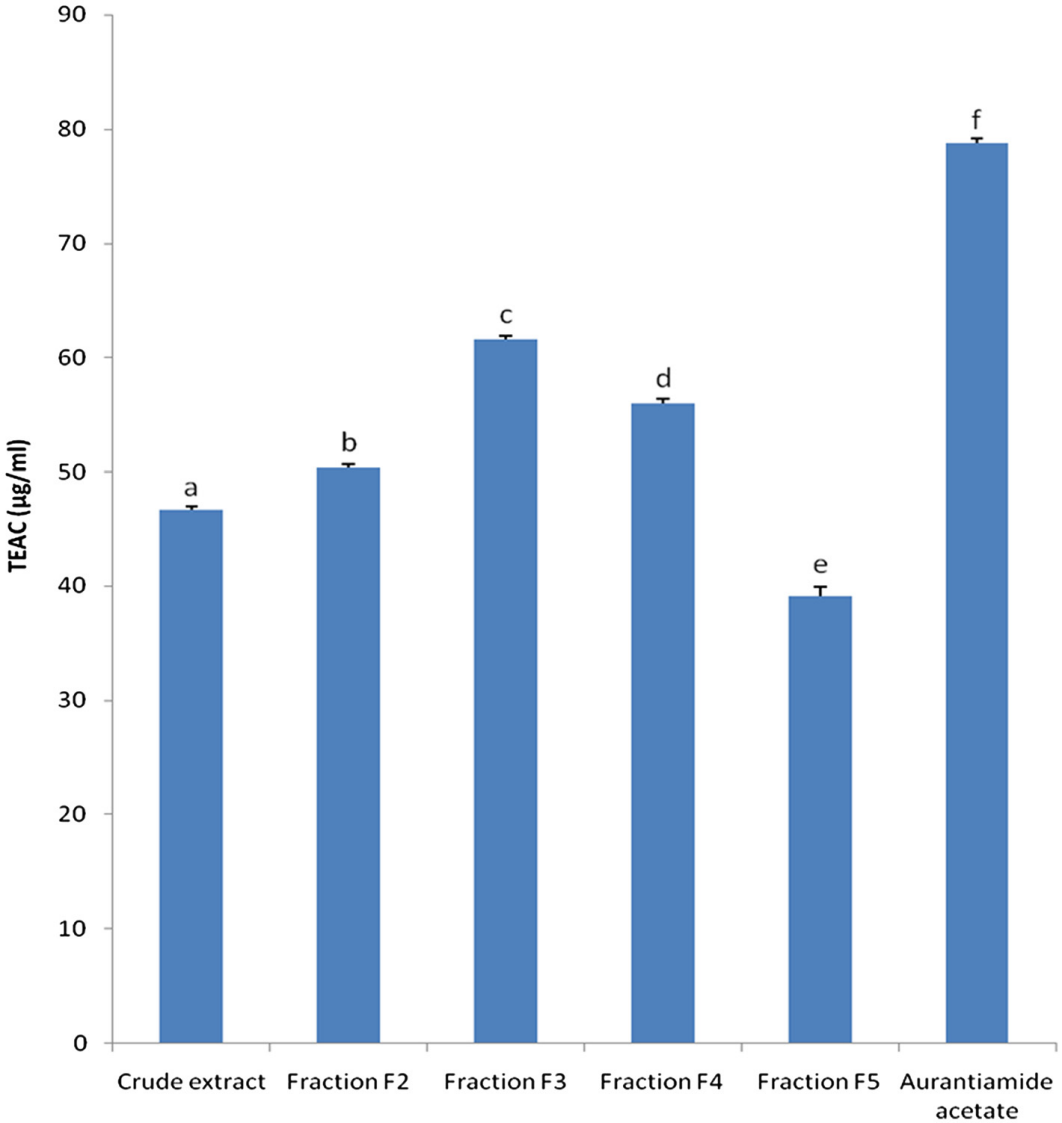
**Gallic acid equivalent antioxidant capacity (TEAC; μg/ml) of tested samples.** Bars represent the mean ± SD of three independent experiments carried out in triplicate. Letters a-f indicate significant differences between samples according to one way ANOVA and Waller Duncan test; *p* < 0.05.

Both with DPPH and TEAC methods, aurantiamide acetate (EC_50_ = 9.51 μg/mL; TEAC = 78.81 μg/mL) showed the highest antioxidant activity (AOA) followed in decreasing order by fraction 3 (EC_50_ = 26.30 μg/mL; TEAC = 61.60 μg/mL), fraction 4 (EC_50_ = 30.11 μg/mL; TEAC = 55.95 μg/mL), fraction 2 (EC_50_ = 32.35 μg/mL; TEAC = 50.32 μg/mL), crude extract (EC_50_ = 70.11 μg/mL; TEAC = 46.72 μg/mL) and fraction 5 (EC_50_ = 77.75 μg/mL; TEAC = 39.10 μg/mL) (Figures 3 and 4). However, the AOA of aurantiamide acetate (EC_50_ = 9.51 μg/mL) was significantly (p<0.05) lower than that of L-ascorbic acid (EC_50_ = 6.81 μg/mL) used as reference antioxidant compound (Figure 3). Fractionation enhanced the AOA of the crude extract in fractions F_2_, F_3_ and F_4_; but this activity was low in fractions F_5_ (Figures 3 and 4). Fractions F_1_ and F_6_- F_9_ were not active (not shown).

### Correlation between the antioxidant capacity and the total phenol content

**Figure 5:**
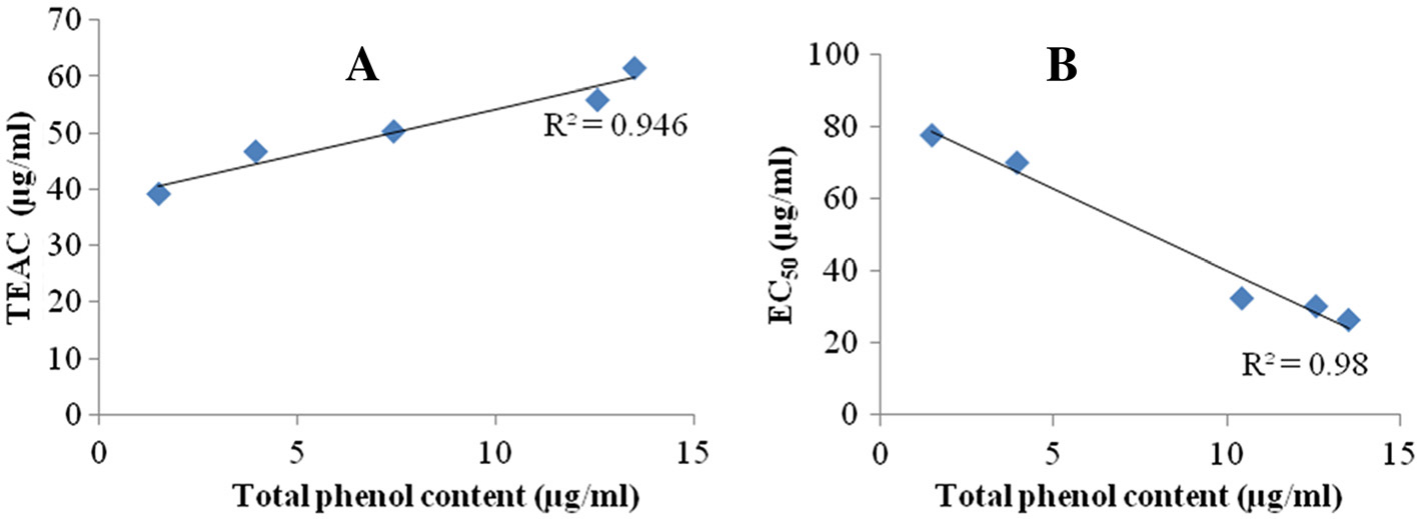
**Positive correlations between the TEAC (A) or EC**_**50**_**(B) and total phenol content.** Results represent the mean ± SD of three independent experiments performed in triplicate. TEAC: gallic acid equivalent antioxidant capacity, EC_50_: equivalent concentration of the test samples scavenging 50% of DPPH radical.

The AOAs were well correlated with the total phenol content: *R*^*2*^ = 0.946 for the TEAC method and *R*^*2*^ = 0.980 for the DPPH free-radical scavenging assay (Figure 5).

## Discussion

The findings of the present study showed that there were differences between the antimicrobial/antioxidant activities of crude extract and those of fractions. Fractions F_2_-F4 (for antioxidant activity) and F_4_ (for antimicrobial activity) were more active than the crude extract indicating that fractionation enhanced the considered biological activities of these fractions. This may be due to the exclusion by fractionation of some constituents of the extract which may tend to dilute the active principle and reduce its activity. On the other hand, fractionation may have increased the concentrations and the activities of antimicrobial/antioxidant principles in these fractions. Our results partially justify the traditional use of this plant for the treatment of skin diseases, bronchitis, typhoid fever and infections of urinary and respiratory tracts that can be caused by the tested microorganisms. The data showed that the response in terms of susceptibility to tested plant extracts/isolated compounds varied among the strains. These variations may be due to genetic differences between the strains. It was also found that MMC values (Table 2) obtained were generally less than four-fold greater than the MICs (Table 1) on the corresponding microbial species, suggesting that the killing effects of the crude extract and the isolated compounds could be expected on most of the tested micro-organisms [13,17,18]. This is very interesting in the perspective of developing antimicrobial drugs from the tested samples. Subfractions B_1_ and B_2_ showed antimicrobial activity against yeasts and bacteria. This is not surprising since some lipid fractions and individual fatty acids have shown this type of biological activities [19,20]. Compounds **1** and **2** displayed both antibacterial and antifungal activities. Comparable results were reported [21,22].

The antioxidant activities of ethyl acetate extract of *A. adianthifolia* stem bark corroborate those reported on the stem bark of *Albizia julibrissin*[23]. Numerous examples of the application of the Folin-Ciocalteu assay to characterize natural products may be found in literature. In most cases, total phenols determined by this method are correlated with the antioxidant capacities confirming the value of the Folin-Ciocalteu test [24]. A new enzymatic method involoving the use of horseradish peroxidase and 4-aminoantipyrine has recently been used in a comparative study of total polyphenol content of tea. Significant differences were found in the results obtained by the Folin-Ciocalteu and enzymatic methods indicating that the enzymatic method needs further standardization [25]. In addition to the compound **2**, phenolic and other nitrogenous compounds were previously isolated from *A. adianthifolia*[4,5]. Phenolic and nitrogenous compounds are known to be potential antioxidant due to their ability to scavenge free radicals and active oxygen species such as singlet oxygen, superoxide anion radical and hydroxyl radicals [26,27]. Therefore, the presence of such compounds could be responsible for the antioxidant activity found in the crude extract and fractions. To the best of our knowledge, this is the first systematic screening for the quantification of phenols and antioxidant activity of the crude extract, fractions and compounds from *A. adianthifolia*.

The overall results of this study can be considered as very promising in the perspective of new drugs discovery from plant sources, when considering the medical importance of tested microorganisms as well as the high level of neurodegenerative diseases associated with oxidative stress. *Pseudomonas aeruginosa* has emerged as one of the most problematic gram-negative pathogens, with the alarmingly high antibiotics resistance rates [28,29]. Even with the most effective antibiotic (carbapenems) against this pathogen, the resistance rates were detected as 15–20.40% amongst 152 *Pseudomonas aeruginosa* strains [29]. This pathogen was found to be sensitive to the crude extract. *Staphylococcus aureus* is a major cause of community and hospital-associated infection with an estimated mortality of around 7-10% [30]. About 77% of immune-deficient patients’ death is attributable to microscopic fungi, such as *Candida* species and *Cryptococcus neoformans*[31]. The prevalence of the typhoid fever caused by *Salmonella typhi* is increased in developing country nowadays [13]. Such findings trace the importance of discovery new substances against which these organisms are sensitive. Generally, at least one sample tested in this study prevented the growth of each microbial strain.

## Conclusions

The results of the present study provide an important basis for use of *A. adianthifolia* in the treatment of oxidative damages and infections associated with the studied microorganisms. The ethyl acetate extract, fractions F2, F3 and F4 as well as aurantiamide acetate found to be the most active samples in this study could be useful for the development of new antimicrobial and antioxidant substances.

## Competing interests

The authors declare that they have no competing interests.

## Authors’ contributions

JDT designated the study, did the extraction/fractionation of the extract and the biological tests under the supervision of JRK. PKL helped to draft the manuscript and in the biological assays. DJMS, MT and PT did the isolation and structure elucidation part and helped in manuscript writing and editing. All authors read and approved the final manuscript.

## Pre-publication history

The pre-publication history for this paper can be accessed here:

http://www.biomedcentral.com/1472-6882/12/99/prepub
